# Letter to the Editor: The death of a neurotrauma trial Lessons learned from the prematurely halted Randomized Evaluation of Surgery in Elderly with Traumatic Acute SubDural Hematoma (RESET-ASDH) trial

**DOI:** 10.1016/j.bas.2024.102916

**Published:** 2024-08-06

**Authors:** Payoz Pandey, Mahendra Pratap Singh, Sanjit Sah

**Affiliations:** Department of Neurosurgery, Graphic Era (Deemed to be University), Clement Town, Dehradun, India; Center for Global Health Research, Saveetha Medical College and Hospital, Saveetha Institute of Medical and Technical Sciences, Saveetha University, Chennai, India; Department of Paediatrics, Dr. D. Y. Patil Medical College, Hospital and Research Centre, Dr. D. Y. Patil Vidyapeeth, Pune, 411018, Maharashtra, India; Department of Public Health Dentistry, Dr. D.Y. Patil Dental College and Hospital, Dr. D.Y. Patil Vidyapeeth, Pune, 411018, Maharashtra, India

Dear Editor,

We are writing in response to the research article titled “The death of a neurotrauma trial: Lessons learned from the prematurely halted Randomized Evaluation of Surgery in Elderly with Traumatic Acute SubDural Hematoma (RESET-ASDH) trial" (Singh et al., 2024). The Randomized Evaluation of Surgery in Elderly with Traumatic Acute SubDural Hematoma (RESET-ASDH) trial was halted prematurely, providing a unique opportunity to reflect on the barriers to conducting neurotrauma trials, especially in elderly populations. We discuss the implications of such barriers and suggests strategies to address them, aiming to guide future trials towards successful completion.

Clinical trials in neurosurgery, particularly those involving critical conditions like traumatic brain injuries in elderly patients, face distinct challenges (Singh et al., 2022). The RESET-ASDH trial's premature termination sheds light on these challenges, highlighting the need for a reevaluation of clinical trial methodologies in high-stakes environments. The principle of clinical equipoise remains a cornerstone of ethical clinical trials, yet its misinterpretation can significantly impede trial progress. In the RESET-ASDH trial, the concept was often confused with personal uncertainty among surgeons, leading to hesitance in patient recruitment (Hartings et al., 2023). Addressing this issue requires clear communication and educational interventions that reinforce the true meaning of equipoise as a state of genuine uncertainty within the expert medical community, rather than individual hesitance.

A recent study examined 52 randomized trials across different clinical fields that were prematurely halted due to futility (Walter et al., 2020). Most of these studies were multi-center, involved drug testing, and about 40% received industry funding. It was common for these trials to have at least one planned interim analysis, generally occurring when approximately half of the intended sample size had been reached. However, only a few of these studies reported low conditional power as a rationale for stopping early, with the conditional power often being low, particularly under the prevailing trend hypothesis.

The COVID-19 pandemic dramatically affected the conduct of clinical trials worldwide (Brown et al., 2023; McDonald et al., 2023), with the RESET-ASDH trial being no exception. Delays in ethical approvals, resource reallocation, and changes in patient availability due to lockdowns underscore the necessity for adaptive trial designs that can withstand external shocks. Future trials should incorporate flexible protocols that can adjust to changing circumstances without compromising scientific integrity or ethical standards.

Elderly patients often have specific concerns and preferences that can complicate their participation in clinical trials (Schwartz et al., 2024), particularly those involving invasive procedures. The RESET-ASDH trial highlighted the importance of considering these preferences and engaging patients and their families through informed, empathetic communication strategies. Building trust and understanding through patient-centered care models and shared decision-making should be integral to trial designs.

To improve the success rate of neurotrauma trials, especially in elderly populations, we recommend the following:

1. **Enhanced Training in Clinical Equipoise:** Educate all trial stakeholders about the ethical underpinnings and practical implications of clinical equipoise.
2. **Robust Contingency Planning:** Develop trial protocols with built-in flexibility to accommodate unforeseen events such as global pandemics.
3. **Patient-Centric Approaches:** Utilize patient engagement strategies that respect individual preferences and conditions, potentially including preliminary surveys or focus groups to tailor trial designs to patient needs.

The lessons from the RESET-ASDH trial are both cautionary and constructive. By understanding and addressing the multifaceted barriers to conducting neurotrauma trials, researchers can better design and execute studies that are not only scientifically rigorous but also ethically sound and responsive to participant needs.

## Declaration of competing interest

The authors declare that they have no known competing financial interests or personal relationships that could have appeared to influence the work reported in this paper.
